# Spatiotemporal variations of ozone exposure and its risks to vegetation and human health in Cyprus: an analysis across a gradient of altitudes

**DOI:** 10.1007/s11676-022-01520-2

**Published:** 2022-08-20

**Authors:** Stefanos Agathokleous, Costas J. Saitanis, Chrysanthos Savvides, Pierre Sicard, Evgenios Agathokleous, Alessandra De Marco

**Affiliations:** 1 The Cyprus Institute, Nicosia, Cyprus; 2 University of the Aegean, Mytilene, Lesvos Greece; 3 Agricultural University of Athens, Athens, Greece; 4 Department of Labour Inspection, Ministry of Labour and Social Insurance, Nicosia, Cyprus; 5 ARGANS, 260 route du Pin Montard, Biot, France; 6 School of Applied Meteorology, Nanjing University of Information Science and Technology (NUIST), Nanjing, 210044 People’s Republic of China; 7 National Agency for New Technologies, Energy and Sustainable Economic Development, Rome, Italy

**Keywords:** Air pollution, Ozone risk assessment, Exposure metrics, Vegetation, Human health

## Abstract

Ground-level ozone (O_3_) affects vegetation and threatens environmental health when levels exceed critical values, above which adverse effects are expected. Cyprus is expected to be a hotspot for O_3_ concentrations due to its unique position in the eastern Mediterranean, receiving air masses from Europe, African, and Asian continents, and experiencing a warm Mediterranean climate. In Cyprus, the spatiotemporal features of O_3_ are poorly understood and the potential risks for forest health have not been explored. We evaluated O_3_ and nitrogen oxides (NO and NO_2_) at four regional background stations at different altitudes over 2014−2016. O_3_ risks to vegetation and human health were estimated by calculating accumulated O_3_ exposure over a threshold of 40 nmol mol^−1^ (AOT40) and cumulative exposure to mixing ratios above 35 nmol mol^−1^ (SOMO35) indices. The data reveal that mean O_3_ concentrations follow a seasonal pattern, with higher levels in spring (51.8 nmol mol^−1^) and summer (53.2 nmol mol^−1^) and lower levels in autumn (46.9 nmol mol^−1^) and winter (43.3 nmol mol^−1^). The highest mean O_3_ exposure (59.5 nmol mol^−1^) in summer occurred at the high elevation station Mt. Troodos (1819 m a.s.l.). Increasing (decreasing) altitudinal gradients were found for O_3_ (NO_x_), driven by summer–winter differences. The diurnal patterns of O_3_ showed little variation. Only at the lowest altitude O_3_ displayed a typical O_3_ diurnal pattern, with hourly differences smaller than 15 nmol mol^−1^. Accumulated O_3_ exposures at all stations and in all years exceeded the European Union’s limits for the protection of vegetation, with average values of 3-month (limit: 3000 nmol mol^−1^ h) and 6-month (limit: 5000 nmol mol^−1^ h) AOT40 for crops and forests of 16,564 and 31,836 nmol mol^−1^ h, respectively. O_3_ exposures were considerably high for human health, with an average SOMO35 value of 7270 nmol mol^−1^ days across stations and years. The results indicate that O_3_ is a major environmental and public health issue in Cyprus, and policies must be adopted to mitigate O_3_ precursor emissions at local and regional scales.

## Introduction

Tropospheric ozone (O_3_) ranks third as a greenhouse gas with regards to radiative forcing contributing to climate change (Myhre et al. 2013). Surface O_3_ is mainly formed through the reaction of carbon monoxide (CO), nitrogen oxides (NO_x_), and volatile organic compounds (VOCs) in the atmosphere under sunlight, in addition to stratospheric O_3_ inputs (Kondratyev and Varotsos 2001). At high concentrations, O_3_ can cause adverse effects on organic and inorganic materials (Screpanti and De Marco 2009) and human health, e.g., cardiovascular and respiratory diseases (Lelieveld et al. 2015; Nuvolone et al. 2018). It can also adversely affect plants and natural ecosystems, resulting in lower yields and productivity (De Marco 2009; Li et al. 2018; Feng et al. 2022; Ryalls et al. 2022), visible foliar O_3_ injury (Sicard et al. 2020a, 2021), a reduction of growth (Proietti et al. 2021), and a decline of biodiversity (Agathokleous et al. 2020a). O_3_ pollution can also contribute to forest decline (Takahashi et al. 2020).

Ground-level O_3_ concentrations, and trends over time, vary spatially and differ from country to country and from region to region (Akritidis et al. 2014; Araminiene et al. 2019; Sicard 2021). The annual O_3_ average concentrations at mid-latitude in the Northern Hemisphere range between 35 and 50 nmol mol^−1^, with the highest levels in the latitude band of 15°−45°N, particularly around the Mediterranean basin (> 50 nmol mol^−1^), while the lowest O_3_ (< 20 nmol mol^−1^) has been recorded in the Southern Hemisphere (Sicard et al. 2017). Compared to urban areas, higher O_3_ concentrations are found in rural areas because of greater emissions of biogenic VOCs, decreased O_3_ titration by NO, and transport of O_3_ and/or precursors from urban areas (Monks et al. 2015; Derstroff et al. 2017; Huang et al. 2018; Yan et al. 2019; Sicard et al. 2020b). O_3_ levels in central Europe show the highest peaks in spring and summer, whereas areas with less air pollution in north or western Europe have peak levels in spring (Cooper et al. 2014). In remote/rural areas, O_3_ peaks occur in spring due to stratospheric inputs as well as precursors accumulated during winter (Sicard et al. 2009; Monks et al. 2015). Since the early 1990s, anthropogenic emissions of O_3_ precursors have been increasing in East and South Asia and decreasing in North America and Europe (Richter et al. 2005; Lamarque et al. 2010; Granier et al. 2011; Xing et al. 2013; Duncan et al. 2016; Zhang et al. 2021). In the Mediterranean basin, the highest concentrations are in the eastern part, with peak concentrations in July, a phenomenon associated with the transfer of O_3_-rich gas masses from the upper troposphere (Doche et al. 2014). Simulations of O_3_ in Europe during 1996−2006 with the RegCM3/CAMx modeling system showed a significant increase of annual O_3_ mean concentrations in the southern UK and Benelux associated with decreased NO_x_ emissions that result in lower O_3_ titration by NO (Akritidis et al. 2014). Conversely, a significant negative trend was found at rural stations in the Mediterranean region due to reduced emissions of O_3_ precursors in Europe (Sicard et al. 2013 and 2016a; Akritidis et al. 2014). On a European scale, the emission of O_3_ precursors decreased over Europe in the period 2000−2014 (27.9% and 35.4% reduction for NO_x_ and NMVOC), associated with O_3_ decreasing by 0.4 nmol mol^−1^ per year (Colette et al. 2011, 2016; Anav et al. 2019). This negative O_3_ trend has been reported to reach 1.1 nmol mol^−1^ per decade in the Mediterranean region (Proietti et al. 2021). Another review of O_3_ trends showed that O_3_ levels decreased in rural areas by 0.24 nmol mol^−1^ per year in North America and by 0.41 nmol mol^−1^ per year in Europe between 2005 and 2014 (Sicard 2021). Conversely, the same study suggested that O_3_ concentrations increased in most cities by 0.33 nmol mol^−1^ per year in North America and by 0.27 nmol mol^−1^ per year in Europe (Sicard 2021). However, in the Northern Hemisphere, O_3_ levels increased by an average of 0.11 nmol mol^−1^ per year at 93 background stations over 1996−2005 (Sicard 2021). This increase can be attributed to higher CH_4_ emissions, changing lightning NO_x_ emissions, and weakened NO titration in a climate change context (Sicard 2021).

Predictions based on current climatic conditions suggest that average O_3_ levels in Europe may decline by about 1 nmol mol^−1^ across Europe by 2050, given that more stringent European air quality policies have been implemented (Hendriks et al. 2016; Anav et al. 2019). A decrease of 3−10 nmol mol^−1^ could be achieved for the hemispheric O_3_ background concentrations around Europe in 2050 if the rest of the world implements such stringent air-quality measures as well (Hendriks et al. 2016). Predictions depend on climate scenarios, and even if O_3_ concentrations decrease, the reductions are numerically small, and O_3_ concentrations are expected to remain at levels considerably higher than pre-industrial levels, and potentially phytotoxic, until 2100 due to climate change (Varotsos et al. 2013; Sicard et al. 2017). Moreover, some representative concentration pathways (RCP) emission scenarios predict increases in O_3_ concentrations by 2100 in sensitive areas (Sicard et al. 2017). Globally, O_3_ concentrations are predicted to increase by 4−5 nmol mol^−1^ in the most pessimistic scenario, RCP8.5, or decrease by 2−10 nmol mol^−1^ by 2100 in the most optimistic scenario, RCP2.6 (Sicard et al. 2017). The largest increases (about 16 nmol mol^−1^) may occur in the southwestern and southeastern Mediterranean because of increased biogenic isoprene emissions under high NO_x_ levels (Varotsos et al. 2013). Furthermore, the eastern Mediterranean and Middle East regions are anticipated to emerge as a “hot spot” of global climate change, while Cyprus may exhibit the most adverse climate change effects by 2100 (Lelieveld et al. 2012).

As average O_3_ concentrations are expected to remain at levels that may threaten living organisms, further studies of O_3_ risks to vegetation are needed. A few studies showed that O_3_ levels in Cyprus are high (e.g., average values often exceeding 40 nmol mol^−1^), while locally produced O_3_ is minor, being affected by transboundary transport due to the unique geographical position of Cyprus as a center point of the Mediterranean Sea (Georgiou et al. 2018; Kleanthous et al. 2014; Kushta et al. 2018; Mallik et al. 2018; Pyrgou et al. 2018a, b). Though Cyprus hosts some of the oldest forests upon which humans have depended since their early settlement about 6000 BC (Ciesla 2004), O_3_ risks to nationwide forest vegetation have never been assessed. Hence, it is important to study local scale O_3_ patterns to better understand O_3_ formation as well as O_3_ risks to forests and other types of vegetation. This study aimed at revealing the space–time patterns of O_3_ and its precursor NO_x_ in Cyprus over the period 2014−2016 and evaluating whether O_3_ is a risk to vegetation and human health. The O_3_ exposure metric of AOT40 (sum of the hourly exceedances above 40 nmol mol^−1^ for daylight hours during the growing season) is used to assess risks to vegetation, while SOMO35 (annual sum of daily maximum 8-h means over 35 nmol mol^−1^) is used to evaluate human health risks. AOT40 is used by regulatory authorities worldwide, while SOMO35 has also been proposed by the European Union (Paoletti et al. 2007; Lupascu and Butler et al. 2019). This study utilizes data from four rural stations to focus on background O_3_, i.e., without the influence of local effects (Snel et al. 2004). Such studies barely exist worldwide because the majority of monitoring stations are found within and around urban areas (Paoletti et al. 2007; Feng et al. 2022). Hence, this study also aimed at elucidating on the effects of elevation on O_3_ and its precursor NO_x_.

## Materials and methods

### Study area

Located in the northeastern part of the Mediterranean Sea, Cyprus is the third largest island in the Mediterranean after Sardinia and Sicily, as well as the third smallest European country after Malta and Luxembourg. The population of the Republic of Cyprus at the end of 2019 was estimated at 888,000 people (Ministry of Finance of the Republic of Cyprus 2020). Cyprus covers an area of 9250 km^2^, 240 km long and 100 km wide and dominated by the Troodos Mountains with Mount Olympus (1951 m a.s.l.) the highest peak. It has a Mediterranean climate, with dry and warm-to-hot summers (May−October), mild winters (November-March), and intermediate transitional seasons, autumn and spring. The weather is sometimes affected by dust originating from desert regions of North Africa and the Middle East, which can influence the diurnal patterns of O_3_ (Mamouri et al. 2016). Based on the Köppen classification of climate, Cyprus’ climate can be classified as both “hot-summer Mediterranean” (Csa) and “hot semi-arid” (BSh).

### Data selection and calculations

**Fig. 1:**
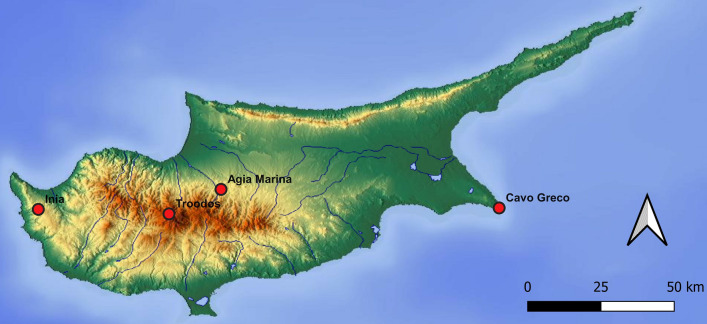
Map of Cyprus illustrating the location of the four rural background stations: Agia Marina (35.02 N−33.03 E, 532 m a.s.l.), Cavo Greco (34.57 N−34.04 E, 23 m a.s.l.), Inia (34.57 N−32.22 E, 672 m a.s.l.), and Troodos (34.56 N−32.51 E, 1819 m a.s.l.). © OpenStreetMap contributors, Creative Commons Attribution-ShareAlike 2.0 license (CC BY-SA 2.0)

Hourly data for NO, NO_2_, and O_3_ were provided by the Air Quality Section of the Department of Labor Inspection (www.airquality.gov.cy) for four background stations for 2014, 2015, and 2016: two inland regional background stations, one high elevation (Troodos, 1819 m a.s.l.) and one mid elevation (Agia Marina Xyliatou, 532 m a.s.l.) included in the European Monitoring and Evaluation Program (EMEP), and two rural background stations in coastal areas (Inia, 672 m a.s.l., and Cavo Greco, 23 m a.s.l.). The four stations are scattered across different areas of the island and cover a range of altitudes, representing risks to vegetation and health in remote and mountainous agricultural areas and forests (Fig. 1).

The stations produced more than 75% of validated hourly data per year. The O_3_ mean concentrations were calculated for different meteorological seasons in the Northern Hemisphere, i.e., winter (December-February), spring (March–May), summer (June–August), and autumn (September–November). Daytime-to-nighttime O_3_ concentration ratios were calculated using the hourly concentrations from 08:00 to 19:00 for the daytime and from 20:00 to 07:00 for night-time. The O_3_ exposure index for vegetation AOT40 (in nmol mol^−1^ h) was calculated as the sum of the hourly exceedances above 40 nmol mol^−1^ for daylight hours during the assumed growing season at our latitude, i.e., 1st April–30th September for the protection of forest trees (UNECE 2010) and 1st May−31st July for agricultural crops (European Union, 2008). Furthermore, considering the climate of Cyprus with numerous trees being physiologically active almost all-year-round, 12-month AOT40 was also calculated with Eq. 1 (Anav et al. 2016), although not used for legislative standards:

1 $$\mathrm{AOT}40={\sum }_{i=1}^{n}{\left(\left[{O}_{3}\right]-40\right).dt} for\left[{O}_{3}\right]>{40\mathrm{ nmol mol}}^{-1}$$

where, [*O*_3_] is hourly O_3_ concentration (nmol mol^−1^) derived from the stations, *n* the number of hours in the calculation period, and *dt* is time step (1 h).

AOT40 is considered as an easy and fast way to determine the presence of O_3_ concentrations potentially affecting vegetation, particularly used when information about plant physiology and other environmental parameters needed to calculate O_3_ fluxes (the actual dose of O_3_ taken up by plants) is not available (Anav et al. 2016; Sicard et al. 2016a; Agathokleous et al. 2018 and 2019). The critical level for the protection of cultivated agricultural crops is set at 3000 nmol mol^−1^ h, which has been adopted by EU legislative bodies (European Union 2008), below which no adverse effects on plants are expected (Agathokleous et al. 2018, 2019). For the protection of forests, a critical level of 5000 nmol mol^−1^ h over the growing season is recommended by UNECE (2010).

SOMO35 is a metric for health impact assessment recommended by the World Health Organization (Malley et al. 2015) and used according to the European guidelines of air quality (Ellingsen et al. 2008). It is widely used in Europe for human health protection, defined as the sum of excess of daily maximum 8-h means (MDA8) over the cut-off concentration of 35 nmol mol^−1^ in a year (Amann et al. 2005; Lupascu and Butler 2019). It was also found to produce lower uncertainty in modeling estimates of the Atmospheric Chemistry and Climate Model Intercomparison Project (ACCMIP) in China compared to standards of the WHO (50 nmol mol^−1^) or China (80 nmol mol^−1^) (Feng et al. 2019). SOMO35 is calculated with Eq. 2 as:

2 $$\mathrm{SOMO}35=\sum_{i=1}^{n }\mathrm{max}({(MDA8}_{i}-35), 0).dt$$

No limit or target value has been set by the EU directives for air quality (Lupascu and Butler et al. 2019); however, a critical level of 3000 nmol mol^−1^ d appears consistent with European air quality limits (Ellingsen et al. 2008; Sicard et al. 2016a).

To test whether O_3_ and its impact metrics depend upon altitude, the parametric Pearson correlation test was applied between the elevations of monitoring stations and each main parameter, i.e., all-year-round daily hourly O_3_ mean concentrations and NO_x_ and the values of human health (SOMO35) and vegetation exposure (3-, 6- and 12-month AOT40) risks. Furthermore, to examine if altitude has a significant effect on O_3_ and NO_x_ concentrations, the hourly averages (*n* = 24) of each station were used. For each hour, the average was calculated as the mean of the four seasonal averages to attribute the same weight to all the seasons. A Box-Cox power transformation (Box and Cox 1964) was then applied to the data according to the procedure described by Agathokleous et al. (2016a). The transformed data were submitted to a general linear model (GLM) adjusted with Overall and Spiegel Method I sum of squares (Howell and McConaughy 1982). The station was a fixed factor, and the hour a random factor. For significant main effects, Tukey’s honestly significant difference (HSD) post hoc comparisons were applied. Data were tested statistically at a level of significance of *a* = 0.05. Data processing and analysis were done with MS Excel (Microsoft) and STATISTICA v.10 (StatSoft Inc. ©) software.

## Results and discussion

### Spatiotemporal distribution of surface O_3_ levels

**Fig. 2:**
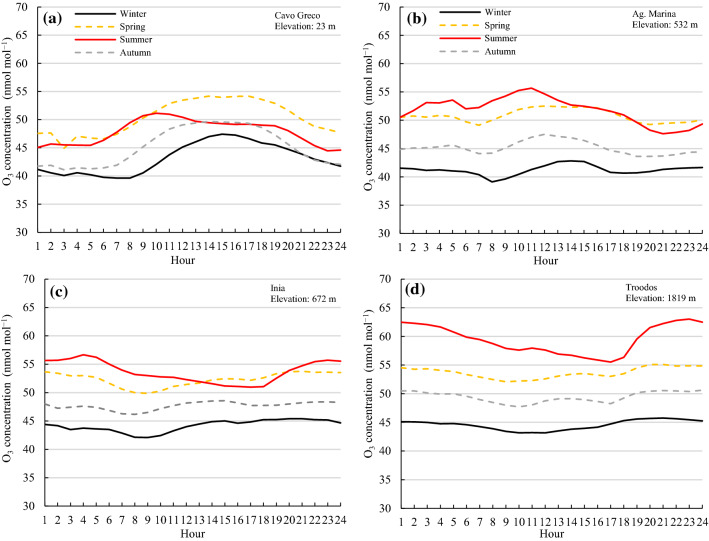
Hourly ozone (O_3_) concentrations averaged over the period of 2014−2016 for the four seasons at **a** Cavo Greco, **b** Agia Marina Xyliatou, **c** Inia, and **d** Troodos monitoring stations

The lowest elevation station (23 m a.s.l.) showed a typical O_3_ diurnal pattern within a range of concentrations of approximately 15 nmol mol^−1^ (Fig. 2a). Diurnal ground-level O_3_ variations depend on the intensity of solar radiation, i.e., O_3_ concentrations are lower during the night because of the absence of photolysis reactions of NO_2_ and photooxidation of CO, VOCs, and other O_3_ precursors (Monks et al. 2015; Sicard et al. 2016a). It also depends on the swift elimination of O_3_ by NO and impeded stratospheric replenishment during the night due to relatively stable temperatures (Monks et al. 2015; Sicard et al. 2016a). During daylight hours, increased O_3_ levels are linked to photooxidation of O_3_ precursors and downward O_3_ transport by convective heating (Derwent et al. 2015; Monks et al. 2015). In rural areas, NO emissions are weaker and nighttime O_3_ can persist at concentrations over 30 nmol mol^−1^ due to no O_3_ titration by NO (Mavrakis et al. 2010; Sicard et al. 2016a). Hence, at these stations, the daytime-nighttime variability in O_3_ was low. Specifically, the ratio of daytime (08:00−19:00) to nighttime (20:00−07:00) ranged from 0.93 to 1.14 across all seasons (Table 1), and the ratios tended to decrease with increasing elevation (*y* =  − 5E−0.5*x* + 1.07; *R*^2^ = 0.70), suggesting lower daytime-nighttime variability as elevation increases.

**Table 1:** Ratio of daytime (08:00−19:00) to nighttime (20:00−07:00) O_3_ concentrations at each background station over the period of 2014−2016

Station	Winter	Spring	Summer	Autumn	All seasons
Cavo Greco	1.079	1.103	1.085	1.135	1.100 ± 0.025*
Ag. Marina	1.000	1.031	1.047	1.025	1.027 ± 0.020
Inia	0.993	0.974	0.939	1.001	0.975 ± 0.028
Troodos	0.975	0.975	0.928	0.971	0.960 ± 0.023

^*^Average ± SD; SD, standard deviation

**Fig. 3:**
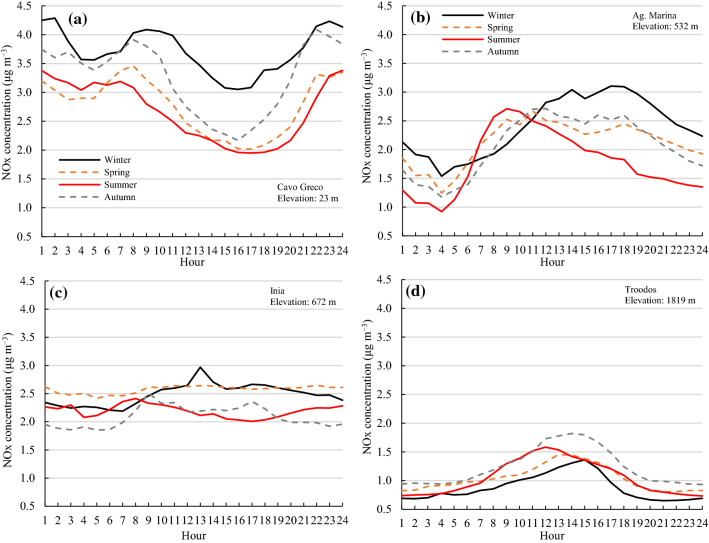
Hourly NO_x_ concentrations (µg m^−3^) averaged over 2014−2016 for the four seasons at **a** Cavo Greco, **b** Agia Marina Xyliatou, **c** Inia, and **d** Troodos monitoring stations

At all stations, the highest seasonal mean O_3_ levels (> 50 nmol mol^−1^) were recorded during late spring and summer, while the lowest (≈ 40 − 45 nmol mol^−1^) were observed during winter (Fig. 2). Higher O_3_ concentrations in spring and summer are mainly because of higher intensity of solar radiation, longer daylight hours, and long-range transport (Kleanthous et al. 2014; Monks et al. 2015; Sicard et al. 2016a; Han et al. 2018; Rizos et al. 2022). Stratospheric O_3_ inputs and biogenic, biomass burning, and lightning NO_x_ emissions are maximum during spring (Lelieveld and Dentener 2000; Han et al. 2018; Zhao et al. 2021). During the cold winters with reduced sunlight, O_3_ titration occurs in the presence of high NOx levels (lack of NO_2_ photolysis reactions), and thus the newly emitted NO reacts spontaneously with O_3_ to produce NO_2_ (Monks et al. 2015; Sicard et al. 2016a). At the low elevation station of Cavo Greco (23 m a.s.l), the highest concentrations of O_3_ in spring could be explained by the proximity of the Vasilikos’ power plant (640 MW capacity). During summer, air conditioning is widely used, leading to higher titration of O_3_ by freshly emitted NO. At this station, about 10% lower hourly NO_x_ concentrations were recorded in summer compared to spring (Fig. 3c). With regards to mid elevation stations, Agia Marina (532 m a.s.l.) and Inia (672 m a.s.l.), the diurnal pattern of O_3_ concentrations was not typical (Fig. 2b, c) and O_3_ levels showed negligible fluctuation over the course of the day, except in summer at the Agia Marina station where concentrations fluctuated within a range of 10 nmol mol^−1^, with peaks during midday hours and minima late night (Fig. 2b). At the Inia station, lower O_3_ concentrations were observed during the daytime; however, the maximum-minimum concentration difference was little, within 5 nmol mol^−1^ (Fig. 2c).

The seasonal O_3_ fluctuation at the Agia Marina station is comparable to the fluctuation during the period 2011−2012 (Kleanthous et al. 2014), but also with that at other agricultural stations in the Eastern Mediterranean (Gerasopoulos et al. 2006; Kalabokas et al. 2008). Several studies in Cyprus show that O_3_ levels differ from region to region and increase during the summer months, with daily O_3_ values ranging from 40 to 100 nmol mol^−1^ (Georgiou et al. 2018; Kushta et al. 2019; Mallik et al. 2018; Pyrgou et al. 2018a, b). Monthly values (April–September) at Agia Marina ranged from 45 to 53 nmol mol^−1^ over the period of 1997−2001 (Kalabokas et al. 2008), and from 40 to 55 nmol mol^−1^ (all months) between 1997 and 2012 (Kleanthous et al. 2014). The value of 40 nmol mol^−1^ is important as it has been proposed as the “safe limit” in the second edition of the WHO’s Air Quality Guidelines for Europe 2000, and is used by regulators in the EU as well as in the U.S for vegetation (Agathokleous et al. 2019).

High elevation sites generally exhibit weaker diurnal variation, with often similar O_3_ levels between day and night, while the day-night pattern can be reversed to show nighttime maxima (Sicard et al. 2009; Karlsson et al. 2021). At the high elevation station of Troodos (1819 m a.s.l.), high hourly O_3_ concentrations exceeding 60 nmol mol^−1^ were observed over the period 2014–2016 (Fig. 2d). At high elevations (> 1200 a.s.l.), the height of the nocturnal mixing layer is minimum. This results in higher O_3_ concentrations by containment (Chevalier et al. 2007). Stations at high elevations can also exceed the planetary boundary layer (Bloomer et al. 2010). The oxidation of terpenes and other VOCs is dominated by reactions with nitrate radicals, affecting the formation of O_3_ (Folkins and Chatfield 2000). NO_x_ modulates the nighttime chemistry of O_3_ via O_3_–NO–NO_2_ interactions (Sicard et al. 2016a; Wang et al. 2022). Higher concentrations are recorded at night due to lower gas-phase titration of O_3_ with NO. These findings confirm that vegetation at high elevations may be at high O_3_ risk (Sicard et al. 2016a, b). Elevated O_3_ concentrations at night are important in determining the response of vegetation to O_3_ due to stomatal opening, especially because high exposures can impair stomatal functioning and keep stomata open longer at night, thus increasing O_3_ influx (Sicard et al. 2016b; Hoshika et al. 2019). Importantly, a recent study also showed that greater plant biomass loss could be caused by equivalent O_3_ fluxes during the nighttime due to the depletion of cell wall-localized ascorbate (Goumenaki et al. 2021). These findings indicate that nighttime O_3_ impacts should be considered in modeling (Wang et al. 2022). In such areas, O_3_ risks to vegetation might be considerably underestimated with current exposure metrics, and nighttime O_3_ impacts should be considered in regional modeling.

**Fig. 4:**
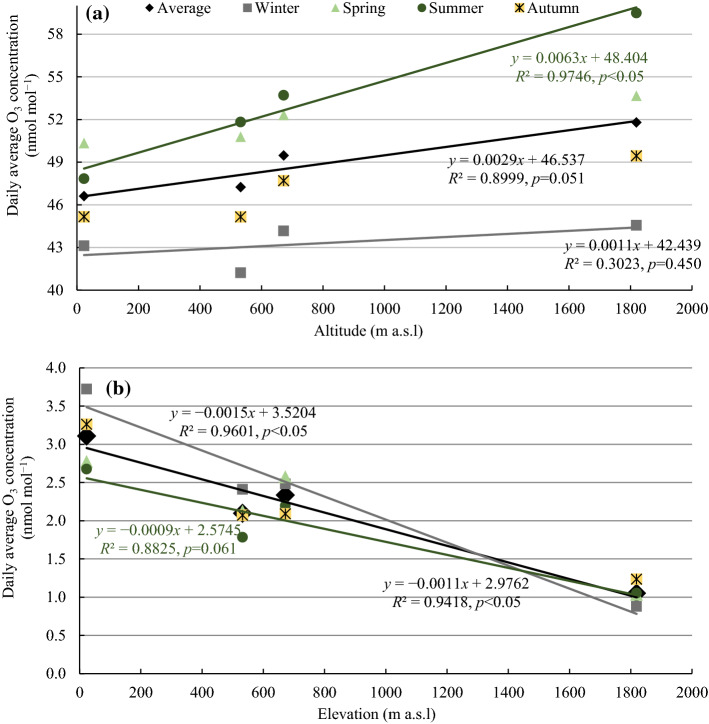
Linear regression between **a** daily O_3_ mean concentrations and **b** daily NO_x_ mean concentrations, averaged over the period 2014−2016, and the elevation (m a.s.l.) of the monitoring stations

Hourly NO_x_ concentrations were considerably low (˂ 5 µg m^−3^) at all stations and in all seasons (Fig. 3). Troodos (1819 m; Fig. 3d) and Inia (672 m; Fig. 3c) stations showed little variation in NO_x_ among seasons and over the course of a day (within 1 μg m^−3^). This variation was higher at Agia Marina (within 2 μg m^−3^) (532 m; Fig. 3b) and Cavo Greco (within 2.5 μg m^−3^) (23 m; Fig. 3a) stations. A distinct pattern in NO_x_ concentrations, with higher levels in winter than in summer at Cavo Greco (Fig. 3a) and Agia Marina (Fig. 3b) stations was observed. Concentrations of O_3_ (Fig. 4a) and NO_x_ (Fig. 4b) were significantly correlated with the elevation of the monitoring station, positively for O_3_ and negatively for NO_x_. The results of regression analysis are further supported by GLM analyses (Table 2). Hence, overall, NO_x_ concentrations decreased and O_3_ concentrations increased with increasing elevation.

**Table 2:** Average (± SE) hourly O_3_ (nmol mol^−1^) and NO_x_ (µg m^−3^) concentrations at each background station over the period 2014−2016

Station	O_3_ (*F* = 49.7; *p* < 0.001)	NO_x_ (*F* = 142.3; *p* < 0.001)
Cavo Greco	93.2 ± 1.09^c^	3.1 ± 0.10^a^
Agia Marina	94.5 ± 0.47^c^	2.1 ± 0.09^b^
Inia	98.9 ± 0.36^b^	2.3 ± 0.02^b^
Troodos	103.6 ± 0.51^a^	1.1 ± 0.05^c^

Data were analyzed with a general linear model followed by Tukey’s honestly significant difference comparisons; different letters indicate statistically significant differences (*p* < 0.05) between stations within a column

The results of NO_x_ concentrations in this study agree with findings in other studies. NO_2_ values over the 2008−2013 period (at a station southeast of Nicosia city) were significantly below the EU permissible values (Mouzourides et al. 2015). Annual averages of NO_x_ concentrations during the same period ranged between 8.5 and 18.0 nmol mol^−1^, which do not exceed the permissible limits (Mouzourides et al. 2015). In another study, it was reported that daily NO_x_ concentrations in July 2014 were below 10 nmol mol^−1^ at the same monitoring stations with the present study, i.e., Cavo Greco, Agia Marina Xyliatou, Inia, and Troodos (Georgiou et al. 2018). In a different study in the summer of 2014, levels were below 0.10 nmol mol^−1^ (Mallik et al. 2018). Similar results were reported in a study conducted in the summer of 2014, where the maximum NO values in Inia were 0.15 nmol mol^−1^ at 09:00 (average = 0.06 nmol mol^−1^), decreasing over the following hours, while NO_2_ levels during the day ranged between 0.10 and 0.20 nmol mol^−1^ (Meusel et al. 2016). An analysis of the daily distribution of NO and NO_2_ concentrations under high temperatures during the 2007–2014 period showed that maximum hourly NO and NO_2_ values were below 12.5–25.0 nmol mol^−1^ (Pyrgou et al. 2018a). More recently, an analysis of data recorded at a station operating on the Campus of the University of Cyprus in the Municipality of Aglantzia, southeast of Nicosia city, revealed that NO_2_ concentrations in 2018 − 2020 were below the levels set by the EU Directive on Air Quality 2008/50/EC (Alexandrou et al. 2021). Hence, NO_x_ levels in Cyprus are relatively low and well within the legal limit (Kushta et al. 2019; Derstrof et al. 2017).

O_3_ concentrations at rural stations are generally 5 − 10 nmol mol^−1^ higher than at urban stations (Kushta et al. 2019). An analysis of urban O_3_ concentrations in Nicosia during the period 2007 − 2014 showed that they exceeded the EU’s limit 79 days, with ≈ 85% occurring during the summer months, 13% in spring, and < 3% in autumn (Pyrgou et al. 2018a and b). O_3_ levels in Cyprus largely depend on the air quality in other European countries due to long-range transport of air masses (Kleanthous et al. 2014; Georgiou et al. 2018). Local emissions of precursors such as NO and CO were responsible for only 6% of the observed O_3_ levels in July 2014 (Georgiou et al. 2018). This finding agrees with that of Kleanthous et al. (2014) that local photochemistry was responsible for only 3 nmol mol^−1^, i.e., about 6% of O_3_ levels, according to analysis of data from the Agia Marina station over the period 1997−2012. These results show that the transport of air masses over long distances (Han et al. 2018) is responsible for much of the recorded O_3_ in Cyprus and cannot be restricted by only local regulations. Thus, O_3_ is expected to be a major environmental problem in the country, with levels that will possibly exceed the permissible limits. It should be noted that ship and aircraft emissions in and over the eastern Mediterranean region around Cyprus are also expected to play an important role in local O_3_ levels (WHO 2008; Marmer and Langmann 2005; Huszar et al. 2010; Koffi et al. 2010). However, a search in the Web of Science with the keywords ‘Cyprus’ AND ‘ozone’ AND ‘ship’ (or ‘aircraft’ or ‘traffic’) revealed no relevant publications (search method: Topic; accessed 15 April 2022), and thus there is no published information about the contribution of maritime shipping and aircraft emissions to the local O_3_ status. It is also important to highlight that local O_3_ formation depends on the VOCs to NO_x_ ratio (Sicard et al. 2020b). However, data for VOCs were unavailable for this study. In areas characterized by high VOCs-NO_x_ ratios (NO_x_-limited regime), a smaller NO_x_ inhibits the formation of O_3_, whereas an increase in VOCs has little or no effect (Sicard et al. 2020b).

### Ozone risks to vegetation and human health

The O_3_ levels reported in this study can be characterized as highly phytotoxic to cultivated crops as the AOT40 is 4−8 times above the critical level of 3000 nmol mol^−1^ h (Table 3) established by the EU. The AOT40 for perennial vegetation (including forests) exceeded by 5−9 times the critical level (i.e., 5000 nmol mol^−1^ h). This result indicates that the AOT40 occurring at rural background stations in Cyprus is considerably higher than the mean AOT40 of 13,718 nmol mol^−1^ h for perennial vegetation based on 3324 global sites (Mills et al. 2018).

**Table 3:** Values of AOT40 (nmol mol^−1^ h) vegetation index at each background station and linear regression between AOT40 values and elevation of the station over 2014−2016

Year	Station	AOT40 (3 months)	AOT40 (6 months)	AOT40 (12 months)
2014	Cavo Greco	12,857	25,080	43,084
	Agia Marina	14,528	26,722	37,596
	Inia	14,135	28,946	44,398
	Troodos	18,337	38,494	52,775
2015	Cavo Greco	14,018	27,263	46,239
	Agia Marina	17,012	30,067	42,303
	Inia	13,238	27,149	44,228
	Troodos	20,572	38,494	50,661
2016	Cavo Greco	14,427	29,813	51,855
	Agia Marina	18,159	33,438	47,080
	Inia	18,695	32,839	51,302
	Troodos	22,795	43,721	61,837
All years*	Cavo Greco	13,767 ± 814	27,385 ± 2,369	47,059 ± 4443
	Agia Marina	16,566 ± 1,856	30,076 ± 3,358	42,326 ± 4742
	Inia	15,356 ± 2,926	29,645 ± 2,909	46,643 ± 4036
	Troodos	20,568 ± 2,229	40,236 ± 3,018	55,091 ± 5937
Linear regression		*y* = 3.72*x* + 13,735 *R*^2^ = 0.94, *p* < 0.05	*y* = 7.41*x* + 26,196 *R*^2^ = 0.96, *p* < 0.05	*y* = 5.58*x* + 43,533 *R*^2^ = 0.63, *p* = 0.206

AOT40 was calculated over 3 months (May−July; legislation for agricultural crops), 6 months (April−September; legislation for forests), and 12 months (non-legislation; more representative for Cyprus forests) per year. *, average ± SD; SD: standard deviation

AOT40 values were higher in 2016 than in 2015 and 2014 for all stations and period of accumulation (3-, 6-, and 12-month). The highest values were recorded at the high elevation station of Troodos (Table 3). In particular, the value for crops (3 months) was on average 33%, 39%, and 33% higher at the Troodos station compared to the other stations in 2016, 2015, and 2014, respectively (Table 3). Similarly, the AOT40 value for forests (6-months) averaged 37%, 37%, and 43% higher at the Troodos station compared to the other stations in 2016, 2015, and 2014, respectively. However, when calculated over the entire year, it was on average 24%, 15%, and 27% higher at Troodos station compared to the other stations in 2016, 2015, and 2014, respectively (Table 3). These results indicate that the difference in AOT40 values between the highest elevation station (Mt Troodos) and the other stations became smaller when calculated over a 12-month period. This may be attributed to the considerably lower O_3_ concentrations in winter and autumn than in summer at Mt Troodos, versus a smaller difference in summer than in winter and autumn and even peaks in spring in lower elevation stations (Cavo Greco, 23 m a.s.l.; Fig. 2). The contribution of stratospheric O_3_ at high-elevation sites is also smaller in the autumn–winter months than in the spring and summer (Lelieveld and Dentener 2000; Terao et al. 2008).

The legislative 3- and 6-month AOT40 for risks to vegetation were significantly correlated (*R*^2^ > 0.94) to the elevation of the monitoring stations (Table 3). Similar to the non-legislative 12-month AOT40, the SOMO35 human health risk index was not significantly correlated to the elevation of the monitoring stations (Table 4), and may be explained by the statistical independence of O_3_ concentrations on elevation in winter (Fig. 4a). Lower O_3_ concentrations, often below 40 nmol mol^−1^ occur in winter, while AOT40 accounts for only hourly concentrations exceeding 40 nmol mol^−1^. These results also suggest that low background O_3_ levels (< 40 nmol mol^−1^) are not correlated to elevation while the higher O_3_ concentrations and peaks are correlated.

**Table 4:** Human health metric: Sum of ozone means over 35 nmol mol^−1^ (SOMO35at each background station and linear regression between SOMO35 and elevation (m a.s.l.) of the station over the period of 2014−2016

Station	SOMO35 (nmol mol^−1^ d)			
	Year			Average ± SD
	2014	2015	2016	
Cavo Greco	7535	6778	7273	7195 ± 384
Agia Marina	6087	6528	6833	6483 ± 375
Inia	7256	7051	7689	7332 ± 326
Troodos	8126	7572	8506	8068 ± 469
Linear regression				y = 0.623*x* + 6795 *R*^2^ = 0.53, *p* = 0.273

SD: standard deviation

The considerably higher AOT40 values at Mt Troodos, compared to the other stations at much lower elevations, demonstrate that both cultivated crops and (semi-) natural vegetation at Mt. Troodos may be more threatened by O_3_. The Troodos Mountains provide habitats to a significant portion of the Cypriot flora, including endemic and endangered plant species that may be at risk, e.g., the Cyprus cedar (*Cedrus brevifolia* A. Henry ex Elwes & A. Henry) (Agathokleous et al. 2015). Therefore, O_3_ may be an additional stressor that threatens endangered plant species. The role of O_3_ as a phytotoxic pollutant has been studied for several decades, and there have been numerous studies demonstrating its negative effects on several cultivated and natural species, including forest trees (Hoshika et al. 2017; Paoletti et al. 2019; Agathokleous et al. 2020a; Sicard et al. 2020a). However, the sensitivity of local plants in Cyprus to O_3_ is unknown (Agathokleous et al. 2015). O_3_ levels escalate during the months that coincide with the growing season of many plants (Saitanis et al. 2020). O_3_ exposures exceeding species-specific thresholds can cause various effects, such as sluggishness or impairment of leaf stomata (Hoshika et al. 2015), visible foliar injury (Calatayud and Cerveró 2007; Moura et al. 2018; Sicard et al. 2020a), growth inhibition (Proietti et al. 2016; Cailleret et al. 2018), reduced resistance to disease, and the ability to compete or coexist with microorganisms (Agathokleous et al. 2016b; Wang et al. 2016). Finally, enhanced O_3_ levels can adversely affect crop production and yield quality, as cultivated plants (e.g., wheat, rice) of high nutritional value to humans and to animals (such as ruminants) are exposed to high concentrations of O_3_ throughout the year during their biological cycle (Malley et al. 2015; Hayes et al. 2016; Li et al. 2018; Feng et al. 2019). Therefore, such high ambient O_3_ exposures may already be harming forest vegetation and crops in Cyprus, suppressing yields and reducing productivity, and contributing to economic losses. For example, annual crop yield losses in East Asia, estimated at US$63 billion, are associated with O_3_ pollution, with China exhibiting the highest relative loss estimated at 9%, 23%, and 33% for maize, rice, and wheat, respectively (Feng et al. 2022). Wheat is a primary staple in Cyprus, and although the sensitivity of local Cyprus wheat to O_3_ is unknown, numerous studies in Asia, Europe, and North America concluded that wheat exhibits among the highest yield losses due to O_3_ (Feng et al. 2022). Feng et al. (2019) also found that critical AOT40 levels were exceeded over 98% and 83% of the forest and wheat areas, respectively, and annual forest biomass and wheat yields were suppressed by 11−13% and 6% in China in 2015. The economic losses of such O_3_ impacts were estimated at US $52.2 billion for forest production and US $11.1 billion for wheat (Feng et al. 2019). More recently, ambient O_3_ pollution was estimated to suppress the annual production of firewood, timber poles, roundwood, and paper pulp by 7.5%, 7.4%, 5.0%, and 4.8%, respectively, and the annual economic damage ranged from 31.6 to 57.1 M€ in Italy (Sacchelli et al. 2021). While O_3_ impacts depend on the sensitivity of local plants under local edaphoclimatic conditions, it cannot be excluded that the O_3_ exposures to key agricultural and forest areas of Cyprus revealed in this study have led to considerable declines in crop and forest productivity, and thus to economic losses. Therefore, further studies are needed to reveal the impacts of O_3_ on Cyprus’s local vegetation and any associated economic damage.

An abundance of studies have indicated that air pollution negatively affects human health (Lelieveld et al. 2015; Li et al. 2018; Feng et al. 2019; Sicard et al. 2021). The effects depend on the type of air pollutant, its concentration, the time and duration of exposure, and the amount that penetrates the lungs (WHO 2006). In addition, some groups of people are more sensitive to air pollution than others, such as pregnant women, the elderly, and people with respiratory problems (WHO 2006). Middleton et al. (2010) reported that children exposed to air pollution in Nicosia have a higher risk of asthma. In line with increasing O_3_ levels in urban areas (Sicard 2021), the annual O_3_-related premature deaths increased in the European Union on average by 0.55 deaths per 1 million (Sicard et al. 2021). Between 2000 and 2017, the highest annual increase was observed in Greece (+ 2.41 deaths per 1 million) while the annual number attributed to O_3_ increased by 0.14 deaths per 1 million in Cyprus (Sicard et al. 2021). In another study, after analyzing O_3_ and air temperature data for the period 2007−2014, Pyrgou et al. (2018a and b) found that O_3_ levels were elevated during heatwaves but were not associated with number of deaths. In contrast, temperature was significantly associated with mortality (Pyrgou et al. 2018b). These studies show that while there is a risk of air pollution to human health in Cyprus, the extent is uncertain, as more studies are needed where multiple factors will be considered, and especially the effect of temperature. However, to our knowledge, no paper has reported SOMO35 in Cyprus before (last search in the Web of Science with the keywords “Cyprus” and “ozone” and “health”, search method: Topic, on 5 March 2022). The average SOMO35 value across stations and years was 770 nmol mol^−1^ d (Table 4), 2.4 times higher than the critical level of 3000 nmol mol^−1^ d (Ellingsen et al. 2008) and almost twice as high than the national average (max = 6074 nmol mol^−1^ d) of rural and suburban Mediterranean stations (located at ≤ 600 m a.s.l.) in Italy in 2000−2004 (Paoletti et al. 2007). The values of SOMO35 found at Cyprus’s background stations in this study however are like those estimated in wide areas of China in 2015 (Feng et al. 2019). A total of 74,000 premature, non-accidental deaths were attributed to O_3_ based on SOMO35 (Feng et al. 2019). Although SOMO35 accounts for acute health effects but not for possible chronic effects of exposures < 35 nmol mol^−1^ (Feng et al. 2019), these results suggest that ambient O_3_ levels in Cyprus may contribute to premature deaths, especially of small sub-populations residing in rural mountainous areas. To this end, further studies are needed to reveal a possible link between SOMO35 and premature mortality in remote populations.

### Potential solutions

Although VOCs have not been included in the present study, they play a crucial role in regulating O_3_ in the atmosphere, and the ratio NO_x_ to VOC is more important than NO_x_ concentrations to regulate O_3_ (Akimoto and Tanimoto 2022; Wang et al. 2022; Zhang et al. 2022). An important point that needs attention is the VOC emissions from vegetation, since a considerable part of the VOC is contributed from vegetation, with plant species showing a wide variability in the size and composition of emissions, even among species of the same family (Richards et al. 2013; Sicard et al. 2018; Fu 2022; Masui et al. 2022). Greening and re‐naturing cities are keywords of the EU Biodiversity Strategy for 2030, calling on European cities of at least 20,000 inhabitants to develop “ambitious urban greening plans”. Therefore, local authorities should consider VOC emissions when selecting tree species for greening strategies in both urban and rural areas (Sicard et al. 2022). Information in this area has increased in recent years and allows for such an application. For example, Sicard et al. (2018) classified 95 plant species based on their ability to optimize air quality and minimize the potential for adverse effects (e.g., allergies). One tactic would be to involve experts specializing in the specific subject (environment-ecology-health) in the first stages of designing such policies, to use the most up-to-date scientific knowledge, and to achieve the best possible result.

Another strategy would be to improve public transport and encourage public use to help reduce locally produced O_3_ precursors. To achieve this, it would be important to raise public awareness, mainly through programs from an early age such as lectures and leaflets in primary and secondary schools. Related to human health, the Air Quality Department publicizes real-time direct air quality information. This is an excellent source of information that can be used to inform the public, especially during periods of high O_3_ levels or in cases of O_3_ episodes to avoid unnecessary exposure and especially outdoor physical exercise.

Kleanthous et al. (2014) reported that at the stations of Inia and Agia Marina, O_3_ concentrations may be reduced by 5.5 nmol mol^−1^ when the winds are from the north, even if the reduced levels are usually less than 1 nmol mol^−1^. The problem of O_3_ in Cyprus may be difficult to alleviate at a national level due to the transnational transport of O_3_ and its precursors. Even if this was not an issue, the mitigation of O_3_ is challenging due to its photochemistry that can lead to increases in concentrations with NO_x_ reduction because NO_x_ is an O_3_ scavenger. This occurrence has been observed worldwide when NO_x_ reductions increased O_3_ concentrations in cities during the COVID-19 pandemic lockdowns (Sicard et al. 2020c; Adam et al. 2021). Hence, with regards the mitigation of O_3_ impacts, coordinated action among governments, the scientific community, and environmental and agricultural agencies are needed at the international level.

However, to assess O_3_ risks on local vegetation, improving the understanding of only O_3_ exposures and spatiotemporal trends and reducing O_3_ exposures is inadequate. The sensitivity of plants to O_3_ is not only species-specific but also highly genotypic-specific (Hayes et al. 2007; Resco de Dios et al. 2016; Mills et al. 2018; Agathokleous et al. 2020a; Yadav et al. 2020; Cotrozzi 2021; Mukherjee et al. 2021, 2022). Therefore, although this study revealed the general risks to vegetation, the actual risks to local species, ecotypes, genotypes, or cultivars would be smaller or higher depending on plant- and condition-specific characteristics. To evaluate specific O_3_ phytotoxicity risk for crop cultivars and wild plants in Cyprus, experimental field O_3_-treatment evaluations, such as with open-top chambers or free-air O_3_-concentration enrichment (FACE) systems are needed (Kobayashi 2015; Paoletti et al. 2016). Alternatively, or in combination, in situ application of antiozonants, e.g., ethylenediurea (EDU), can provide important insights into O_3_ impacts on agricultural crops and forests in remote areas without accessible electricity or research infrastructure (Paoletti et al. 2009; Manning et al. 2011; Singh et al. 2015; Tiwari 2017; Saitanis and Agathokleous 2021). Moreover, biomonitoring, using a combination of O_3_-sensitive and O_3_-resistant paired genotypes concurrently (with or without EDU) can provide an additional means to record O_3_ phytotoxicity potential (Harmens et al. 2015; Agathokleous et al. 2020b). Plant materials of such genotypes are available by specific institutions upon request. These efforts would be further substantiated with comprehensive in situ observations of O_3_ symptomology on cultivated crops on local farms as well as on natural vegetation (Harmens et al. 2015; Marzuoli et al. 2019). Cooperation among the scientific community, local authorities, and farmers would facilitate the appropriate establishment of such projects, with the goal to feed models with empirical data to derive specific exposure- and flux-response relationships and effect estimates for Cyprus in the future. Such projects would also be facilitated by the inclusion of a citizen-science approach, for example, by involving military personnel located nearby the monitoring/experimental plots or people living in surrounding areas (Agathokleous et al. 2020b). This is important in recognition of the technical difficulties in maintaining and conducting such projects in remote areas that are not easily accessible, especially for the application of antiozonants that requires repeated treatments throughout the growing season (Paoletti et al. 2009; Manning et al. 2011; Singh et al. 2015; Tiwari 2017; Saitanis and Agathokleous 2021). Involving the public in such programs would also promote environmental education and enhance awareness of environmental issues (Agathokleous et al. 2020b).

A question that should arise is from which plants/taxa to begin. Plant taxa are affected differently by O_3_, depending on the exceedance of their specific detoxification thresholds for damage (Emberson 2020). There is also considerable difference in the sensitivity to O_3_ among functional groups of plants (Agathokleous et al. 2020a; Grulke and Heath 2020), and examination of relevant literature can provide general conclusions for the basis of plant selection that may be at higher O_3_ risk. For example, deciduous species are more sensitive than evergreen, including oaks that are widely distributed and important in Cyprus (Cotrozzi 2021). Oak species native to Eurasia were also found to be more sensitive than oaks from North America (Cotrozzi 2021). Woody species with high leaf dry mass per unit leaf area (LMA) show high sensitivity to O_3_ (Feng et al. 2018). LMA is widely available for a vast array of species, if not, it can be easily measured in situ without special equipment or technique. Thus, LMA may be used as a proxy of potentially more sensitive species that could be prioritized for research. Moreover, modern wheat cultivars may be more sensitive to O_3_ due to breeding for higher yields without factoring in resistance to O_3_ (Saitanis et al. 2014; Singh et al. 2018; Hansen et al. 2019; Yadav et al. 2020). Therefore, not only does this require experimentation, but it also suggests that breeding programs by public agricultural institutions should incorporate resistance to O_3_.

It is important to consider that O_3_ pollution can damage plants without visible injuries and can degrade the quality of edible plants. For example, studies reveal that grape (*Vitis vinifera* L.) can have a 20−30% decreased yield while the quality may have 15−23% less polyphenols, including cultivars such as Cabernet Sauvignon and Merlot (Ascenso et al. 2021; Blanco-Ward et al. 2021). These are major cultivars with cultural and economic importance in Cyprus as well, often cultivated at higher elevations where O_3_ levels are high. Therefore, studies are needed to assess how O_3_ levels affect the quality of edible plant products.

## Conclusion

It is important to study spatiotemporal trends to better understand O_3_ formation and air quality in relation to other EU Member States. O_3_ is a major environmental and public health problem in Cyprus, with levels far exceeding the “permissible safety limits” set by the EU. The present analysis of atmospheric quality at four rural background stations across Cyprus showed that the Troodos Mountains, where many endemic plant species grow, are at high O_3_ risk. Therefore, policies that need to be taken to reduce concentrations of tropospheric O_3_ are urgent. AOT40 assumes that O_3_ concentrations below 40 nmol mol^−1^ and nighttime exposures are negligible, and thus appears inadequate for a realistic quantification of O_3_ impacts on vegetation. Further studies are needed to estimate O_3_ risks to Cypriot vegetation using flux-based approaches that will incorporate nighttime exposure as well.
